# Association between Recipient IL-15 Genetic Variant and the Prognosis of HBV-Related Hepatocellular Carcinoma after Liver Transplantation

**DOI:** 10.1155/2017/1754696

**Published:** 2017-10-15

**Authors:** Tao Zhang, Yuan Liu, Xu Peng, Junwei Fan, Zhihai Peng

**Affiliations:** ^1^Department of Hepatobiliary Pancreatic Surgery, Shanghai General Hospital, School of Medicine, Shanghai Jiao Tong University, Shanghai, China; ^2^Department of General Surgery, Hainan Cancer Hospital, Haikou, China

## Abstract

**Objective:**

To investigate the association of donor and recipient IL-15 genetic variants with HCC recurrence and prognosis after LT.

**Methods:**

A total of 112 liver transplant patients with HBV-related HCC were enrolled. IL-15 rs10519613 and rs13122930 were genotyped in donors and recipients.

**Results:**

Recipient IL-15 rs10519613 polymorphism was found to be significantly related to HCC recurrence after LT. In multivariate analysis, tumor thrombus, UCSF criteria, and recipient IL-15 rs10519613 genotypes were independent predictive factors of HCC recurrence after LT. Kaplan-Meier survival analysis demonstrated that patients with recipient IL-15 rs10519613 CA/AA genotypes had a decreased disease-free survival and overall survival than those with the CC genotype. Recipient IL-15 rs10519613 genetic variant could improve survival prediction when combined with the UCSF criteria. Furthermore, Cox proportional hazard regression analysis revealed that tumor size (*p* = 0.012, *p* = 0.623), tumor thrombus (*p* = 0.011, *p* = 0.015), UCSF criteria (*p* = 0.471, *p* = 0.013), and recipient IL-15 rs10519613 genotype (*p* = 0.039, *p* = 0.008) were independent factors of predicting DFS and OS.

**Conclusions:**

Recipient IL-15 rs10519613 polymorphism was associated with HCC recurrence after LT and might be a potential genetic marker for the clinical outcome of HCC patients treated with LT.

## 1. Introduction

Hepatocellular carcinoma (HCC) is one of the most common malignancies and the third leading cause of cancer-related mortality worldwide [1, 2]. Liver transplantation provides an effective therapeutic option for HCC and the underlying liver cirrhosis. However, HCC recurrence after LT is a serious complication and negatively affects patient survival.

Clinical parameters such as alpha-fetoprotein (AFP), histologic grade, tumor numbers, tumor size, microvascular invasion, tumor thrombus, tumor stage (TNM), and the selection criteria for LT (Milan or UCSF criteria) have been reported as risk factors for HCC recurrence and prognosis after LT [3–8]. Although several criteria are available for transplant patient selection, they have limitations for determining clinical outcome. Molecular markers and genetic variants could provide supplemental and useful information for predicting the clinical outcome and improve the selection of patients for adjuvant therapies after LT in addition to the clinical factors [9, 10]. As such, it is a challenge to identify patients who are with a greater risk for HCC recurrence after LT and potential biomarkers for prognosis prediction.

IL-15 (interleukin 15) encodes the cytokine that is a four-*α*-helix-bundle cytokine and plays a pivotal role in the proliferation and survival of T lymphocytes, NK cells, NKT cells, and different subsets of innate lymphoid cells. More specifically, IL-15 increases the cytotoxicity of CD8^+^ T cells and is essential for NK cell activation and thus is involved in Th1-type immune response, including antitumor response [11–16]. Accumulated evidences have shown that IL-15 was significantly associated with a decreased incidence of tumor recurrence and a prolonged overall survival, including HCC [17, 18]. It is well known that, besides the inflammatory response, some other factors including the genetic variants also play an important role in the expression and activity of cytokines [19]. Several studies have revealed that the genetic variants of a cytokine gene were associated with HCC and HCC recurrence [10, 20, 21].

However, the influence of the IL-15 gene polymorphisms on the prognosis of HCC after LT has not been investigated. In this study, we selected two potentially significant SNPs of IL-15 and aimed to investigate whether donor and recipient IL-15 polymorphisms were associated with HCC recurrence and prognosis after LT in a Han Chinese population.

## 2. Patients and Methods

### 2.1. Patients

A total of 112 hepatitis B virus- (HBV-) related HCC patients who underwent orthotopic liver transplantation between July 2007 and June 2014 at Shanghai Jiao Tong University Affiliated Shanghai General Hospital, China, were enrolled. There were 102 males and 10 females with a mean age of 50.1 ± 8.6 years (range: 21–67 years). The main clinicopathological characteristics of the study population are summarized in Table 1. All patients were routinely followed up at the posttransplant clinic according to our previous study [22], and the mean follow-up time was 38.3 ± 27.9 months (range: 1–105 months).

### 2.2. Ethics Statement

Liver grafts were obtained from donation after cardiac death (DCD) as well as from living related donors. In our center, almost all liver grafts were obtained from DCD; only one liver graft was obtained from LD in the study. Informed consent was obtained from all subjects. The research was approved by the Ethics Committee of Shanghai Jiao Tong University. The methods were carried out in accordance with the Declaration of Helsinki and its later amendments or comparable ethical standards.

### 2.3. Data Collection

The following data were collected before LT: patient age, gender, underlying liver disease (HBV), and preoperative AFP level (<400 ng/ml or ≥400 ng/ml). After LT, tumor size and number, histologic grade (differentiated: well/moderate; undifferentiated: poor), tumor thrombus, pathological TNM staging, cirrhotic background, disease-free survival (DFS), and overall survival (OS) were recorded. The diagnosis of HCC was confirmed by two pathologists. Each tumor in the explant liver was reevaluated to give a judgment according to the UCSF criteria (one tumor of ≤6.5 cm or a maximum of three tumor nodules each of ≤4.5 cm and the sum of tumor diameters of ≤8 cm) on the basis of pathological data [23].

### 2.4. Genomic DNA Isolation and Genotyping

The two potentially significant SNPs (rs10519613 and rs13122930) of IL-15 were selected by our exon array, the International HapMap Project database, and the dbSNP database (a minor allele frequency > 0.05 in Asian populations). Genomic DNA was extracted from the donor and recipient liver tissue (which had been previously stored at −80°C) using an AllPrep DNA/RNA Mini Kit (Qiagen, Hilden, Germany). Genotyping of IL-15 rs10519613 and rs13122930 was conducted using the Sequenom MassARRAY SNP genotyping platform (Sequenom, San Diego, CA, USA) [24]. The protocols included polymerase chain reaction amplification, shrimp alkaline phosphatase treatment, single-base extension reaction, resin cleanup, nanodispensing on SpectroCHIP, and data acquisition. To confirm the genotyping results, >10% of the samples were randomly selected and regenotyped with 100% concordance.

### 2.5. Statistical Analysis

Statistical analysis was performed using SPSS version 19.0 (IBM Corp., Armonk, NY, USA) and GraphPad Prism Version 5.00 software (GraphPad Inc., La Jolla, CA, USA). The Hardy-Weinberg equilibrium and the allele frequency were analyzed using SHEsis software [25]. Quantitative variable data were compared using Student's *t*-test, and categorical variables were analyzed by the chi-square test or Fisher's exact test. All variables were evaluated by univariate logistic regression analysis. Variables with statistical significance in univariate analysis were included in the multivariate analysis. Disease-free survival (DFS) and overall survival (OS) between different groups were performed with Kaplan-Meier survival curves and compared using the log-rank test, and variables with significance in univariate Cox proportional hazard regressions were performed in the multivariate analysis. Two-tailed *p* values of less than 0.05 were considered statistically significant.

## 3. Results

### 3.1. Clinicopathological Characteristics of LT Patients

The overall incidence of HCC recurrence after LT was 33.9% (38/112) in this study population. The clinicopathological characteristics in the recurrence and nonrecurrence groups are shown in Table 1. Tumor size (*p* < 0.001), tumor thrombus (*p* < 0.001), TMN stage (*p* = 0.003), and UCSF criteria (*p* = 0.002) were significantly associated with HCC recurrence after LT. However, no significant difference was observed between the two groups concerning other clinicopathological characteristics, including the recipient age, gender, cirrhotic background, histologic grade, tumor number, and serum AFP level.

### 3.2. Effect of IL-15 rs10519613 and rs13122930 Genotypes on HCC Recurrence

Genotype distribution of donor and recipient IL-15 rs10519613 and rs13122930 and the effect of the two SNPs on HCC recurrence after LT are shown in Table 2. The observed SNPs were in accordance with the Hardy-Weinberg equilibrium (*p* > 0.05). No significant linkage disequilibrium between the two SNPs was observed. The incidence of HCC recurrence was significantly higher for patients with recipient IL-15 rs10519613 CA/AA genotypes than for those with the CC genotype (*p* < 0.001), and patients with the recipient IL-15 rs10519613 A allele had higher HCC recurrence than those with the C allele (*p* < 0.001). However, donor IL-15 rs10519613 and donor and recipient IL-15 rs13122930 polymorphisms were not significantly associated with HCC recurrence in our study.

### 3.3. Univariate and Multivariate Analyses for Risk Factors for HCC Recurrence

Univariate and multivariate analyses were used to determine the association between the risk factors and HCC recurrence (Tables 3 and 4). In univariate analysis, tumor size (*p* < 0.001), tumor thrombus (*p* < 0.001), TMN stage (*p* = 0.004), UCSF criteria (*p* = 0.002), and recipient IL-15 rs10519613 genotypes (CA/AA versus CC, *p* = 0.001) were significantly associated with an increased risk of HCC recurrence. All significant factors (*p* < 0.05) identified by univariate analysis were included in a multivariate analysis. Tumor thrombus (OR = 3.591, 95% CI: 1.438–8.971, *p* = 0.006), UCSF criteria (OR = 3.922, 95% CI: 1.515–10.152, *p* = 0.005), and recipient IL-15 rs10519613 genotypes (CA/AA versus CC; OR = 5.143, 95% CI: 1.636–16.168, *p* = 0.005) were independent predictive factors of HCC recurrence.

### 3.4. Survival Analysis

**Figure 1:**
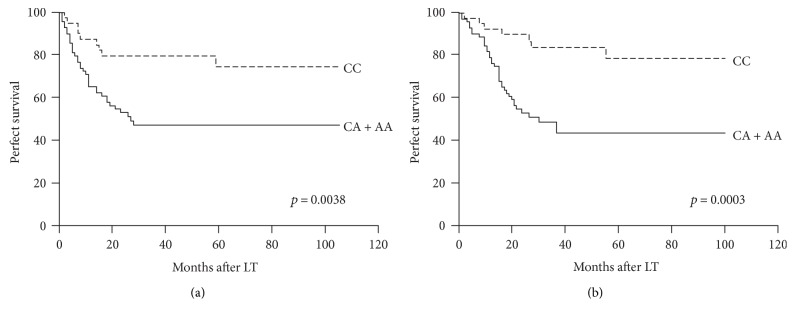
Disease-free survival (a) and overall survival (b) of transplant HCC patients stratified by recipient IL-15 rs10519613 genotypes.

Univariate Cox regression analysis revealed that histologic grade, tumor size, tumor thrombus, TMN stage, UCSF criteria, recipient IL-15 rs10519613 genotypes (CA/AA versus CC) were significantly associated with DFS and OS (Table 5). A Kaplan-Meier plot of patient survival is shown in Figures 1(a) and 1(b). The group of patients with recipient IL-15 rs10519613 CA/AA genotypes had a reduced DFS (log-rank; *p* = 0.0038; Figure 1(a)) and OS (log-rank; *p* = 0.0003; Figure 1(b)) than the group with the recipient IL-15 rs10519613 CC genotype.

The results of multivariate analysis showed that tumor size (OR = 2.213, 95% CI: 1.188–4.122, *p* = 0.012), tumor thrombus (OR = 2.385, 95% CI: 1.224–4.647, *p* = 0.011), and recipient IL-15 rs10519613 genotypes (CA/AA versus CC; OR = 2.214, 95% CI: 1.041–4.708, *p* = 0.039) were independent factors of predicting DFS, whereas tumor thrombus (OR = 2.302, 95% CI: 1.173–4.517, *p* = 0.015), UCSF criteria (OR = 2.209, 95% CI: 1.181–4.131, *p* = 0.013), and recipient IL-15 rs10519613 genotypes (CA/AA versus CC; OR = 3.152, 95% CI: 1.358–7.315, *p* = 0.008) were independent factors of predicting OS (Table 6).

### 3.5. Improved Survival Prediction after Integrating Recipient IL-15 rs10519613 Genetic Factor into the UCSF Criteria

**Figure 2:**
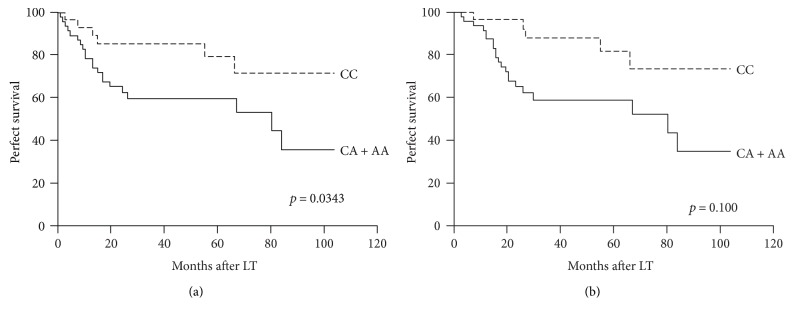
Disease-free survival (a) and overall survival (b) of transplant HCC patients within the UCSF criteria stratified by recipient IL-15 rs10519613 genotypes.

**Figure 3:**
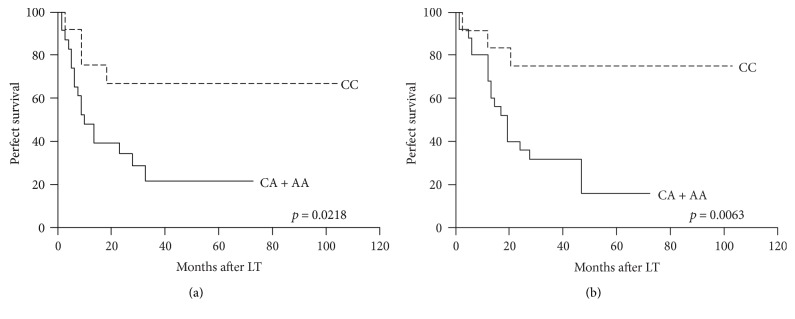
Disease-free survival (a) and overall survival (b) of transplant HCC patients beyond the UCSF criteria stratified by recipient IL-15 rs10519613 genotypes.

To investigate whether recipient IL-15 rs10519613 genetic variant could improve survival prediction when combined with the UCSF criteria, survival differences between the patients with tumors within or beyond the UCSF criteria stratified by recipient IL-15 rs10519613 genetic variant were analyzed. For patients who met the UCSF criteria, recipient IL-15 rs10519613 genotypes (CA/AA versus CC) could independently predict DFS (*p* = 0.0343, Figure 2(a)) and OS (*p* = 0.0100, Figure 2(b)). And for patients who did not meet the UCSF criteria, significant differences in DFS (*p* = 0.0218, Figure 3(a)) and OS (*p* = 0.0063, Figure 3(b)) between the two genotype groups were also observed.

## 4. Discussion

Although some researches have reported the association of IL-15 with the clinical outcome of HCC, studies of the genetic variants in the IL-15 gene on HCC recurrence and prognosis after LT are few. In this study, the overall frequency of IL-15 rs10519613 CC, CA, and AA genotypes were 33.5%, 47.8%, 18.7%, respectively; our data were similar to that of the previous study in other population [26]. Our results revealed that recipient IL-15 rs10519613 polymorphisms (CA/AA versus CC genotype; A versus C allele) were significantly associated with HCC recurrence after LT. In multivariate analysis, recipient IL-15 rs10519613 genotypes (CA/AA versus CC) were independent predictive factors of HCC recurrence after LT. Furthermore, we found that patients with recipient IL-15 rs10519613 CA/AA genotypes had a decreased DFS and OS than patients with the CC genotype in univariate and multivariate Cox proportional hazard regression analyses.

To our knowledge, this is the first study to demonstrate that recipient IL-15 rs10519613 genetic variant influences HCC recurrence and prognosis after LT at the genetic level. IL-15 is a proinflammatory cytokine with similar functions to interleukin 2 (IL-2), including antitumor activity. Given its pivotal role in the proliferation, survival, and activation of CD8^+^ T cells and NK cells, IL-15 has been identified as an antitumor cytokine in several cancers, including neuroblastoma, HCC, and breast and colorectal cancer [27–31]. A close relationship between cytokine gene polymorphisms and HCC and HCC prognosis has been reported in recent years [10, 20, 21]. The potential association between the IL-15 gene polymorphisms and HCC recurrence and prognosis has been described in several studies. A recent study has shown that IL-15 protein levels in peritumoral liver tissues are significantly associated with a decreased incidence of HCC recurrence and a prolonged overall survival [17]. Furthermore, the genetic constitution is related to the level and function of cytokines and thus affects the antitumor immunity of the host. Several reports have demonstrated that IL-15 gene polymorphisms were significantly related to cancer risk and overall survival [32, 33]. Moreover, the A allele of IL-15 rs10519613 has been identified as a risk allele for MRD-positive status that is the most prognostic value for risk assessment in ALL [34].

In addition to the genetic factor, clinical parameters (tumor thrombus, UCSF criteria) were other independent factors influencing the clinical outcome of HCC patients after LT, which were consistent with the previous studies [3, 35–37]. When combining the genetic factor (recipient IL-15 rs10519613 polymorphism) and UCSF criteria to predict the clinical outcome after LT, we found that recipient IL-15 rs10519613 genetic variant was able to stratify transplant patients within or beyond the UCSF criteria into more homogenous groups with distinct DFS and OS after LT. These results suggested that recipient IL-15 rs10519613 polymorphism could discriminate the clinical outcome of HCC patients within or beyond the UCSF criteria and might affect follow-up and therapeutic strategies after LT.

There were several limitations in our study. Firstly, these results were obtained from a relatively small number of Chinese patients. Therefore, larger sample size is warranted to confirm our findings. Secondly, the biological function of IL-15 rs10519613 polymorphism was not performed in this study. Assessment of the functionality of this SNP associated with the clinical outcome of HCC after LT is needed in further studies.

In summary, our results demonstrated that recipient IL-15 rs10519613 genetic variant was an independent predictive factor of HCC recurrence after LT, and integration of this novel genetic marker into the UCSF criteria was helpful for improving the accuracy of prognostication. Therefore, these findings might help to tailor postoperative management strategies for patients with a higher risk of HCC recurrence after LT.

## Figures and Tables

**Table 1:** Demographic data for recurrence and nonrecurrence groups.

	Recurrence group (*n* = 38)	Nonrecurrence group (*n* = 74)	*p* value
Recipient age (yr)	48.47 ± 7.95	50.93 ± 8.82	0.152
Recipient male/female (*n*)	35 (92.1)/3 (7.9)	67 (90.5)/7 (9.5)	0.783
Cirrhosis yes/no (*n*)	30 (78.9)/8 (21.1)	65 (87.8)/9 (12.2)	0.214
Histologic grade (*n*)			0.203
Differentiated	28 (73.7)	62 (83.8)	
Undifferentiated	10 (26.3)	12 (16.2)	
Tumor size (*n*)			<0.001
<5 cm	16 (42.1)	57 (77.0)	
≥5 cm	22 (57.9)	17 (23.0)	
Multinodular yes/no (*n*)	19 (50.0)/19 (50.0)	28 (37.8)/46 (62.2)	0.217
Tumor thrombus (*n*)			<0.001
Yes	24 (63.2)	19 (25.7)	
No	14 (37.8)	55 (74.3)	
Serum AFP level at LT (*n*)			0.489
<400 cm	27 (71.1)	57 (77.0)	
≥400 cm	11 (28.9)	17 (23.0)	
Tumor stage (*n*)			0.003
I-II	22 (57.9)	62 (83.8)	
III	16 (42.1)	12 (16.2)	
UCSF criteria in/out (*n*)	18 (47.7)/20 (52.3)	57 (77.0)/17 (23.0)	0.002

**Table 2:** Comparison of genotype and allele distribution between recurrence and nonrecurrence groups.

SNP (allele)	Genotype distribution (*n*)			*p* value	HWE *p* value
	Genotype	Recurrence	Nonrecurrence		
Donor					

rs10519613	CC	8	27		0.219

(C/A)	CA	22	36	0.132	
	AA	8	11	0.139	
	CC	8	27		
	CA/AA	30	47	0.095	
	C	38	90		
	A	38	58	0.122	

rs13122930	TT	11	13		0.441

(T/C)	TC	19	41	0.221	
	CC	8	20	0.198	
	TT	11	13		
	TC/CC	27	61	0.165	
	C	41	67		
	A	35	81	0.218	

Recipient					

rs10519613	CC	5	35		0.072

(C/A)	CA	21	28	0.002	
	AA	12	11	0.001	
	CC	5	35		
	CA/AA	33	39	<0.001	
	C	31	98		
	A	45	50	<0.001	

rs13122930	TT	9	13		0.294

(T/C)	TC	18	31	0.738	
	CC	11	30	0.252	
	TT	9	13		
	TC/CC	29	61	0.440	
	T	36	57		
	C	40	91	0.203	

SNP: single-nucleotide polymorphism; HWE: Hardy-Weinberg equilibrium.

**Table 3:** Univariate logistic regression analysis of risk factors associated with tumor recurrence.

Risk factors	*p* value	Odds ratio (95% CI)
Tumor size		
(0: <5 cm, 1: ≥5 cm)	<0.001	4.610 (1.987–10.695)
Tumor thrombus		
(0 = no, 1 = yes)	<0.001	4.962 (2.141–11.501)
Tumor stage (*n*)		
(0 = I-II, 1 = III)	0004	3.758 (1.537–9.174)
UCSF criteria (*n*)		
(0 = no, 1 = yes)	0.002	3.758 (1.615–8.595)
Recipient IL-15 rs10519613 genotype		
(0 = CC, 1 = CA/AA)	0.001	5.923 (2.082–16.849)

CI: confidence interval.

**Table 4:** Multivariate logistic regression analysis of risk factors associated with tumor recurrence.

Risk factors	*p* value	Odds ratio (95% CI)
Tumor thrombus		
(0 = no, 1 = yes)	0.006	3.591 (1.438–8.971)
UCSF criteria (*n*)		
(0 = no, 1 = yes)	0.005	3.922 (1.515–10.152)
Recipient IL-15 rs10519613 genotype		
(0 = CC, 1 = CA/AA)	0.005	5.143 (1.636–16.168)

CI: confidence interval.

**Table 5:** Univariate analysis of prognostic factors associated with DFS and OS.

Prognostic factors	DFS (months)		OS (months)	
	Odds ratio (95% CI)	*p* value	Odds ratio (95% CI)	*p* value
Histologic grade				
(0 = differentiated, 1 = undifferentiated)	2.093 (1.119–3.915)	0.021	2.194 (1.162–4.139)	0.015
Tumor size (0: <5 cm, 1: ≥5 cm)	3.238 (1.818–5.768)	<0.001	3.253 (1.791–5.910)	<0.001
Tumor thrombus (0 = no, 1 = yes)	3.893 (2.101–7.015)	<0.001	3.791 (2.035–7.060)	<0.001
Tumor stage (0 = stages I-II, 1 = stage III)	3.276 (1.818–5.904)	<0.001	3.124 (1.695–5.756)	<0.001
UCSF criteria (0 = no, 1 = yes)	2.769 (1.556–4.927)	0.001	2.813 (1.552–5.097)	0.001
Donor IL-15 rs10519613 genotype				
(0 = CC, 1 = CA/AA)	2.267 (1.096–4.690)	0.027	1.778 (0.878–3.601)	0.110
Recipient IL-15 rs10519613 genotype				
(0 = CC, 1 = CA/AA)	3.000 (1.447–6.219)	0.003	4.005 (1.776–9.030)	0.001

DFS: disease-free survival; OS: overall survival; CI: confidence interval.

**Table 6:** Multivariate analysis of prognostic factors associated with DFS and OS.

Prognostic factors	DFS (months)		OS (months)	
	Odds ratio (95% CI)	*p* value	Odds ratio (95% CI)	*p* value
Tumor size (0: <5 cm, 1: ≥5 cm)	2.213 (1.188–4.122)	0.012	—	0.632
Tumor thrombus (0 = no, 1 = yes)	2.385 (1.224–4.647)	0.011	2.302 (1.173–4.517)	0.015
UCSF criteria (0 = no, 1 = yes)	—	0.471	2.209 (1.181–4.131)	0.013
Recipient IL-15 rs10519613 genotype				
(0 = CC, 1 = CA/AA)	2.214 (1.041–4.708)	0.039	3.152 (1.358–7.315)	0.008

DFS: disease-free survival; OS: overall survival; CI: confidence interval.

## Abbreviations

LT: Liver transplantation
IL-15: Interleukin 15
HCC: Hepatocellular carcinoma
AFP: Alpha-fetoprotein
NK cells: Natural killer cells
HBV: Hepatitis B virus
DFS: Disease-free survival
OS: Overall survival
SNP: Single-nucleotide polymorphism
MRD: Minimal residual disease
ALL: Acute lymphoblastic leukemia.
